# Impact and Problems of Fetal Echocardiography: A Single-Institution Study in Japan

**DOI:** 10.7759/cureus.31423

**Published:** 2022-11-12

**Authors:** Kosuke Yonehara, Kazuya Terada, Mikio Morine

**Affiliations:** 1 Pediatrics, Shikoku Medical Center for Children and Adults, Zentsuji, JPN; 2 Pediatric Cardiology, Shikoku Medical Center for Children and Adults, Zentsuji, JPN; 3 Obstetrics and Gynecology, Shikoku Medical Center for Children and Adults, Zentsuji, JPN

**Keywords:** hypoplastic left heart syndrome, total anomalous pulmonary venous conncection, prenatal diagnosis of congenital heart disease, pediatric cardiology, fetal echocardiography

## Abstract

Background: The purpose of this study was to assess the current status of prenatal diagnosis and the benefit of fetal echocardiography for complex congenital heart disease (CHD) in Kagawa prefecture.

Method: We reviewed 152 cases of CHDs who were born between 2012 and 2020 and performed cardiovascular surgery or catheter intervention in our hospital. They were divided into two groups: the fetal diagnosis group (FD) and the no-fetal diagnosis group (nFD). We compared patient data between FD and nFD groups.

Results: Of the 152 patients, 63 patients were FD group and 89 patients were nFD group. The fetal detection rate was lower than 40% between 2012 and 2014, its ratio raise to about 50% after 2015. The ratio of poor general conditions at admission was significantly higher in the nFD group (p<0.01). When focused on hypoplastic left heart syndrome, the number of patients with poor general condition at admission was significantly small in the FD group. Although, the first operation day of life and neonatal death did not show a significant difference between both groups. In total anomalous pulmonary venous connection (TAPVC) cases, 14 cases out of 15 had not been diagnosed prenatally. Among them, the number of cases with poor general condition was nine (64%) and inappropriate usage of oxygen or nitric oxide was 10 cases (71%).

Discussion/conclusion: This study suggests that prenatal diagnosis significantly reduces poor general condition at admission. Although the prenatal detection rate grew up, that of TAPVC remains low and their clinical course is poor. In addition, inappropriate therapies were performed. It is desirable to increase the detection rate of TAPVC.

## Introduction

Congenital heart disease (CHD) accounts for 40% of all congenital fetal anomalies [1]. In recent years, with the increasing popularity of fetal echocardiography, the number of diagnosed prenatal cases of CHD has increased [2]. CHD has a variety of clinical courses. In duct-dependent CHD, neonates require prostaglandin E1 (PGE1) agents soon after birth to prevent ductal shock or hypoxia. In transposition of the great arteries (TGA) or total anomalous pulmonary venous connection (TAPVC), neonates show severe desaturation and may require emergent surgical repair or balloon atrial septostomy (BAS). Thus, prenatal diagnosis of severe CHD is crucial to rescue neonates and reduce their emergent transfer. If CHD is diagnosed prenatally, medical staff can prepare instruments and drugs for treatment initiation immediately after birth. CHD is associated with chromosomal abnormalities such as trisomy 21 and trisomy 18. If chromosomal abnormalities are suspected during the fetal period, genetic counseling can be provided to parents.

In Kagawa Prefecture, Japan, our hospital is the only institution that has a pediatric cardiovascular surgery department; thus, neonates with CHD are often transferred for surgical repair or catheter intervention. In such circumstances, questions arise regarding the prenatal detection ratio of CHD in our hospital and whether fetal echocardiography contributes to a better clinical course.

Here, we assessed the current status of prenatal diagnosis and the benefit of fetal echocardiography for complex CHDs in Kagawa Prefecture, Japan.

## Materials and methods

This was a retrospective study conducted at the Shikoku Medical Center for Children and Adults; the only institution that has a fetal echocardiography department in the Kagawa Prefecture, Japan. We reviewed 152 patients with CHD who were born between 2012 and 2020. CHDs that needed interventions, such as PGE1, cardiac catheterization, or palliative cardiac surgery, in the neonatal period were included. Simple CHDs such as atrial septal defect (ASD), ventricular septal defect (VSD), and other minor anomalies were excluded. The clinical course, gestational age, birth weight, fetal diagnosis, and other information were obtained from the medical records. We divided the patients into two groups: the fetal diagnosis group (FD) and the no-fetal diagnosis group (nFD). We compared patient data between the FD and nFD groups.

Statistical analysis

All data are expressed as mean±standard error. All statistical analyses were performed using the EZR software (https://www.jichi.ac.jp/saitama-sct/SaitamaHP.files/statmed.html). Statistical comparisons were performed using the Mann-Whitney U-test and Chi-square test. Statistical significance was set at P < 0.05.

## Results

Out of the 152 patients studied, 63 and 89 were in the FD and nFD groups, respectively. Demographic data are shown in Table 1. Patients in the FD group were diagnosed at 31.1±4.3 weeks gestational age. Gestational age and weight at birth and the ratio of chromosomal anomalies showed no significant differences between the two groups.

**Table 1:** Demographic data GA: Gestational Age; FD: Fetal Diagnosis; nFD: non Fetal Diagnosis

	FD group(63)	nFD group(89)	p-value
GA of FD (weeks)	31.1±4.3		
GA of delivery (weeks)	38.0±2.0	38.1±1.9	0.67
Body weight (g)	2788±540	2765±635	0.9
Chromosomal anomalies	9	21	0.25

The CHDs and their fetal diagnosis ratios are shown in Table 2. Only one complex case of coarctation of the aorta (CoA) had been misdiagnosed as VSD prenatally. Simple CoA and TAPVC had a low detection rate. In contrast, tricuspid atresia and VSD with pulmonary atresia had high detection rates.

**Table 2:** The number of FD group and nFD group in each CHD and % of prenatal detection rate FD: Fetal Diagnosis, nFD: non Fetal Diagnosis, CHD: Congenital Heart Disease, cAVSD: complete atrioventricular septal defect, TOF: tetralogy of Fallot, HLHS: hypoplastic left heart syndrome, TA: tricuspid atresia, TAPVC: total anomalous pulmonary venous connection, TGA: transposition of great arteries, CoA: coarctation of the aorta, VSD: ventricular septal defect

	FD group (63)	nFD group (89)	FDrate [%]
cAVSD	4	13	23
TOF	12	14	46
VSD+PA	5	1	83
HLHS	4	4	50
TA	5	1	86
TAPVC	1	14	7
TGA (1+2)	7	6	54
Simple CoA	0	5	0
CoA complex (VSD+CoA)	6	12	33
Others	19	19	

**Figure 1:**
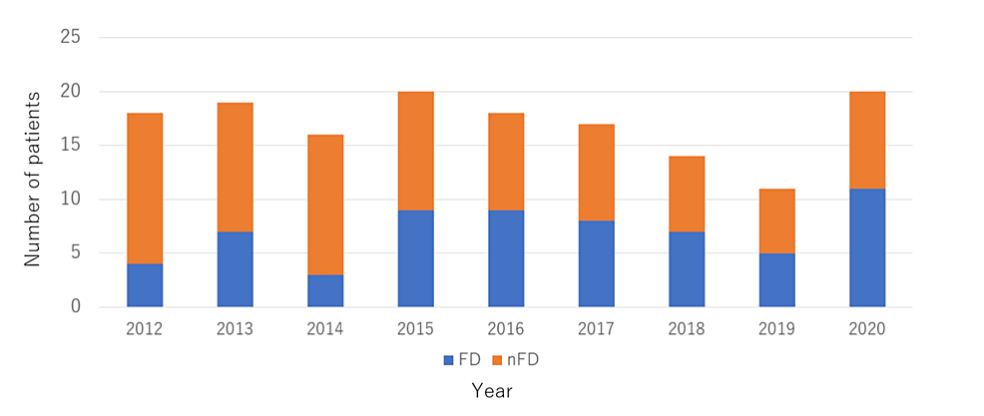
Yearly number of FD and nFD groups Before 2014, the prenatal detection ratio of CHD was lower than 40%. After 2015, the prenatal detection rate raised up to about 50%. FD: Fetal diagnosis; nFD:non-Fetal diagnosis; CHD: congenital heart disease

Figure 1 illustrates the yearly alterations in the number of patients with CHD who underwent surgeries at our hospital. The total number of patients per year ranged from 15 to 20. Although the fetal detection rate was < 40% between 2012 and 2014, it increased to approximately 50% after 2015.

Table 3 shows the clinical course of neonates in the FD and nFD groups. The incidence of poor general conditions at admission was significantly higher in the nFD group than in the FD group (p<0.01). The poor general condition includes ductal shock, high pulmonary arterial flow, and severe hypoxia. There was a significant difference in the use of PGE1 agents; PGE1 agents were used more in the FD group than in the nFD group. With regard to the necessity of neonatal intervention, the age at presentation and neonatal death did not show significant differences between the groups.

**Table 3:** Clinical course of total cases FD group showed significantly low poor general condition and high usage of prostaglandin E1 (PGE1). Other items have no significant differences between both groups.

	FD(63)	nFD(89)	p-value
Poor general condition	2	25	<0.01
Usage of PGE1	34	23	<0.01
The number of neonatal intervention	41	48	0.26
Neonatal death	5	4	0.54
Age of intervention (days)	13.6±7.3	12.9±8.1	0.85

We focused on hypoplastic left heart syndrome (HLHS) and the results are shown in Table 4. Birth weight and gestational age did not differ significantly between the groups. The timing of diagnosis in the nFD group was 1.2±0.5 days. The number of patients with poor general conditions at admission was significantly lower in the FD group than in the nFD group. There was no significant difference on the first postoperative day between the groups.

**Table 4:** Clinical data of HLHS Focused on HLHS, poor general condition at admission was significantly low in the FD group. However, neonatal death was not significantly low in the FD group. HLHS: hypoplastic left heart syndrome, FD: fetal diagnosis, nFD: non-fetal diagnosis

	FD(4)	nFD(4)	p-value
GA of delivery (week)	38.3±0.5	39.0±0.8	0.24
Body weight (g)	2953±293	2716±243	0.11
Age for diagnosis (day)	0	1.2±0.5	0.02
Poor general condition	0	4	0.02
Age for operation (day)	7.0±3.7	7.5±2.0	0.77
Neonatal death	2	2	1.0

Subsequently, we focused on TGA, and the results are shown in Table 5. TGA types 1 and 2 were included. No significant differences were observed in the poor general condition at admission, use of PGE1 agents, the necessity of BAS, day of life during BAS or Jatene operation, and neonatal death between the two groups.

**Table 5:** Clinical data of TGA (type 1+2) No significant differences were observed between both groups. GA: gestational age; TGA: transposition of great arteries; BAS: balloon atrial septostomy; FD; fetal diagnosis; nFD: non-fetal diagnosis

	FD(7)	nFD(6)	p-value
GA for delivery (weeks)	39.2±1.0	38.2±2.4	0.57
Body weight (g)	3107±304	2833±493	0.35
Age for diagnosis (days)	0	0.8±2.0	0.35
Poor general condition	0	1	0.46
Usage of PGE1	6	5	1.0
BAS	3	2	1.0
Age for BAS (days)	2.5±3.5	4.3±5.8	1.0
Age for operation (days)	9.5±4.1	8.3±2.2	0.65
Neonatal death	0	0	1.0

Table 6 shows the TAPVC results. Statistical analysis for TAPVC was impossible because of the small number of cases in the FD group (only one case). Fourteen patients were not diagnosed prenatally. In the nFD group, nine (64%) patients had poor general condition, and oxygen or nitric oxide was inappropriately used in 10 (71%).

**Table 6:** Clinical data of TAPVC More than half of nFD group cases showed poor general condition on admission and inappropriate usage of O_2_/NO_2_. GA: gestational age; O_2_: oxygen; NO_:_ nitric oxygen; TAPVC: total anomalous pulmonary venous connection; FD: fetal diagnosis; nFD: non-fetal diagnosis

	FD(1)	nFD(14)
GA (weeks)	37.4	38.8±1.5
Body weight (g)	2224	2829±344
Median day for diagnosis (days)	0	1.5
Poor general condition	0	9
Usage of O_2_/NO	0	8
The number for neonatal operations	1	10
Age for neonatal operation (day)	3	8.3±10.2
Neonatal death	0	0

## Discussion

In this study, the prenatal detection rate of all complex CHDs increased to 50%. The reason why fetal detection rates have increased is that screening for fetal heart diseases has spread widely, especially among obstetrics practitioners after 2015. Simple CoA and TAPVC are often difficult to detect prenatally because several obstetricians use the normal 4-chamber view as a screening tool. The intracardiac structure in CoA and TAPVC is almost normal and assessing aortic isthmus or pulmonary venous return routinely takes time and effort. Neonatal transfer of postnatally diagnosed CHD is stressful for both babies and medical staff. In particular, severe CHDs such as TAPVC, HLHS, and TGA without fetal diagnosis have a severe clinical course, such as severe hypoxia and ductal shock.

Comparable to previous studies, this study suggests that prenatal diagnosis significantly reduces the poor general condition at admission [3]. PGE1 usage is significantly higher in the FD group that the nFD group because CHDs tend to have abnormal ductus and intracardiac structures, which enables easy detection during prenatal screening.

Satomi et al. reported that prenatal diagnosis of HLHS was beneficial for preventing ductal shock and maintaining the patients’ preoperative condition [2]. In our study, HLHS had a prenatal detection rate of 50%. Four HLHS cases diagnosed prenatally maintained a good general condition before the operation. The FD group in this study did not show a better neonatal postoperative course than the nFD group did. Surgical outcomes of HLHS are poor in Japan. Although various modifications have been attempted by pediatric cardiovascular surgeons, a major factor affecting surgical outcomes is the patient’s preoperative condition. The preoperative condition worsens if ductal shock, renal failure, or necrotizing enteropathy occurs, which can result in poor postoperative outcomes [2].

Focusing on TGA, this study revealed no significant difference in the poor general condition on admission, day of life at the time of Jatene operation, and neonatal death between the FD and nFD groups. Nagata et al. reported that prenatal TGA diagnosis significantly shortened the time interval from birth to neonatal care and surgery, and was associated with improved survival [4]. In our study, the average time from birth to diagnosis in the nFD group was very short (0.8±2.0 day). Kagawa is the smallest prefecture in Japan, with an area of 1,876 km^2^. Owing to its small area, neonates with CHD can be transferred smoothly, which reduces the delay in postnatal diagnosis or therapies.

Despite the small area, postnatally diagnosed TAPVC showed a poor clinical course. More than half of the patients had poor general conditions on admission due to severe hypoxia. In addition, oxygen or nitric oxide was inappropriately used in more than half of the cases, which worsened the pulmonary venous obstruction. TAPVC is often misdiagnosed as persistent pulmonary hypertension of the newborn (PPHN). PPHN presents with dyspnea and desaturation, and its clinical symptoms are similar to those of TAPVC. However, PPHN treatment differs from that of TAPVC. Methods to reduce pulmonary hypertension in PPHN include intubation and oxygen or nitric oxide use. However, these methods worsen pulmonary venous obstruction and hypoxia in patients with TAPVC. Thus, prenatal TAPVC diagnosis can help avoid inappropriate therapy application and improve preoperative conditions.

Limitations of this study

There are some limitations in this study. Firstly, this is a retrospective study and clinical courses were obtained from past medical records. However, medical records were well documented, and correct data were obtained. Secondly, the number of cases was small. As written above, Kagawa is the smallest prefecture in Japan and has a low population density. In our hospital, fetal echocardiography started before 2011. However, medical records before 2011 were unavailable, thus the sample size was small. Thirdly, the number of patients who were diagnosed with TAPVC prenatally was small, which preclude statistical analysis.

## Conclusions

Herein, we present the fetal echocardiography experience in Kagawa Prefecture, Japan. Although the detection rate of total CHDs has recently increased, that of simple CoA and TAPVC remains low. In particular, the prenatal detection rate of TAPVC is < 10%, and the clinical course of TAPVC without prenatal diagnosis is poor. We established that a prenatal diagnosis improved the general condition of the neonate on admission in all CHDs. Furthermore, the postoperative clinical course of HLHS is not satisfactory; thus, further improvements in the surgical technique for HLHS are required. CHD screening should be widely used to increase the fetal diagnosis rate of TAPVC.
